# Correlation between gastrointestinal hormones and anxiety-depressive states in irritable bowel syndrome

**DOI:** 10.3892/etm.2013.1211

**Published:** 2013-07-09

**Authors:** BAOJUAN HAN

**Affiliations:** Department of Gastroenterology, Xingtai People's Hospital, Xingtai, Hebei 054031, P.R. China

**Keywords:** irritable bowel syndrome, gastrointestinal hormones, vasoactive intestinal peptide, somatostatin, anxiety-depression

## Abstract

The aim of this study was to investigate the mechanism(s) of action of gastrointestinal hormones in the pathogenesis of irritable bowel syndrome (IBS), and the correlation between gastrointestinal hormones and psychological factors. Patients with IBS were divided into IBS with normal emotional state ratings and IBS in anxiety-depressive states groups. The two groups were then subdivided into IBS-constipation predominant (IBS-C) and IBS-diarrhea predominant (IBS-D) groups. Non-IBS patients with normal depression and anxiety ratings were recruited as controls. The serum concentrations of somatostatin (SS) and vasoactive intestinal peptide (VIP) were measured by radioimmunoassay, and the expression of SS and VIP in the colonic mucosa was detected by immunohistochemistry and radioimmunoassay. The anxiety-depression scores of patients with IBS were significantly different from those of the control group (P<0.05). The expression levels of SS and VIP in the serum and colonic mucosa of the patients with IBS were higher compared with those of the control group. Furthermore, the expression level of SS in the IBS-C group demonstrated a significantly larger increase than that in the IBS-D group (P<0.05); however, there was no significant difference in the expression of VIP between the IBS-C and IBS-D groups (P>0.05). In addition, the expression levels of SS and VIP in the IBS groups with normal emotional state ratings were notably different from those in the IBS groups in anxiety-depressive states (P<0.05). Anxiety-depressive states may lead to changes in the secretion of SS and VIP, and subsequently to changes in gastrointestinal motility and function.

## Introduction

Irritable bowel syndrome (IBS) is a continuous or intermittent disease characterized by abdominal pain and bloating, and abnormal bowel habits and stool consistency, in the absence of obvious morphological changes or a symptom complex with biochemical abnormalities. IBS occurs in 10–20% of individuals worldwide, 35% of whom find it necessary to visit the doctor (1). To date, the pathogenesis and etiology of IBS remains unclear. At present, IBS is considered to be a heterogeneous disease that occurs due to a variety of factors, in which psychological, spiritual, social and environmental elements are important (2). The process of IBS is controlled by the nervous, immune and endocrine systems, which may lead to intestinal smooth muscle motility disorders and visceral paresthesia. Intestinal motility disorders, which are characterized by high intestinal sensitivity and reactivity, are the direct mechanism for the symptoms of IBS.

In recent years, with developments in the field of nerve gastroenterology, numerous studies have attempted to investigate the correlation between psychological and social factors and the pathogenesis of IBS, and the brain-intestine interaction. The importance of psychological factors in IBS has been indicated by the results of anatomical examinations, including the observation that the emotional center, the autonomic center dominating gastrointestinal tract motility and secretion, and the endocrine regulation center are located at the same anatomic location. The physiological functions of the gastrointestinal tract are performed under the complex and sophisticated regulation of different nervous systems. The nerve-endocrine network connecting the gastrointestinal tract and the central nervous system is known as the brain-intestine axis, while the transmission of gastrointestinal tract activity information to the central nervous system, and the regulation of gastrointestinal tract activity by the central nervous system, is known as the brain-intestine interaction (3). The influence of psychological factors on gastrointestinal tract motility and feeling may be implemented via the neuroendocrine-immune network, while psychosocial stimuli affect the physiological function of body by the evocation of an emotional response (or psychological stress). This emotional response affects respiration, blood pressure, heart rate, smooth muscle movement, glandular secretion, hormone levels, skeletal muscle movement and external behavior performances, through the cerebral cortex, limbic system, hypothalamus, pituitary and the target gland system of the body. The hyperesthesia that results from psychological factors in turn aggravates the psychological and emotional reactions, and forms a feedback loop. The sensitivity of internal organs to sensory stimuli is therefore increased, resulting in the occurrence and aggravation of IBS. In the current study, the interaction between gastrointestinal hormones and psychological factors in IBS was investigated.

## Subjects and methods

### Subjects

Patients with IBS who visited the gastroenterology outpatient department of Xingtai People's Hospital (Xingtai, China) from March 2008 to September 2010 were recruited for this study, and were assigned to groups according to Rome III Diagnostic Criteria, IBS subtype and Self-Rating Anxiety Scale (SAS) and Self-Rating Depression Scale (SDS) scores. The Rome III Diagnostic Criteria for IBS includes symptoms of recurrent abdominal pain or discomfort apparent for at least six months, and experienced on at least three days in the previous three months, with the involvement of at least two of the following: i) pain relief following a bowel movement; ii) an interrelation between the onset of pain and a change in the stool frequency; and iii) an interrelation between the onset of pain and a change in the appearance of the stool. In the classification of IBS subtypes, cases in which >25% of the stools appeared hard or lumpy, and <25% appeared loose or watery were classified as constipation predominant IBS (IBS-C), and cases in which >25% of the stools appeared loose or watery and <25% appeared hard or lumpy were classified as diarrhea predominant IBS (IBS-D). Furthermore, the exclusion criteria comprised: i) organic diseases, such as ulcers, erosion and masses, as demonstrated by endoscopy; ii) organic diseases of the liver, gallbladder, pancreas and intestines, as demonstrated using ultrasound, X-ray and other laboratory tests; iii) diabetes, mental illness, and heart, thyroid and connective tissue diseases; iv) administration of cathartic, antidiarrheal, prokinetic and digestive enzyme drugs within the previous one month; and v) a history of abdominal surgery. The SAS and SDS scores were used to assess the anxiety-depressive states of the patients with IBS (4,5). The patients that met the previously mentioned IBS criteria were divided into four groups: i) IBS-C with normal SAS and SDS scores (n=13), which comprised five males and eight females with an average age of 40.5 years (range, 18–63 years); ii) IBS-C in anxiety-depressive states (n=17), which included six males and 11 females with an average age of 37.5 years (range, 16–59 years); iii) IBS-D with normal SAS and SDS scores (n=12), which included six males and six females with an average age of 39 years (range, 17–61 years); and iv) IBS-D in anxiety-depressive states (n=16), which comprised six males and 10 females with an average age of 37.5 years (range, 17–58 years). Non-IBS patients with normal anxiety and depression ratings, as diagnosed by medical examination, were recruited as a control group. The controls included a total of 15 patients (six males and nine females) who had an average age of 38.5 years (range, 20–57 years) and who were diagnosed with colonic polyps or internal hemorrhoids, following admission to the outpatients unit of Xingtai People's Hospital. No gastrointestinal symptoms, such as abdominal pain, diarrhea or constipation, no neurological symptoms or signs and no recent medical history were reported near to the time of the experiment. This study was approved by Ethics Committee of Xingtai People's Hospital. Informed consent was obtained from all patients.

### Sampling

Fasting venous blood (2 ml) was drawn from each patient early in the morning, and was subsequently added to a test tube containing 30 μl 10% disodium EDTA and 40 μl aprotinin. This was then mixed at 4°C. Following centrifugation at 3,000 rpm (centrifugal radius, 10 cm) for 10 min, the plasma was separated and stored in two test tubes at −20°C for later use. Prior to determination, the plasma sample was defrosted at room temperature, and then centrifuged at 3,000 rpm (centrifugal radius, 10 cm) for 5 min, in order to obtain the supernatant. The sigmoid colon mucosa was added into the homogenizer, followed by 0.1 N acetic acid. Subsequent to full homogenation, the homogenate liquid was transferred to a plastic tube, and left to stand overnight. Following this, an equal volume of 0.1 N NaOH solution was added to the homogenate liquid, and the liquid was then centrifuged at 3,000 rpm for 15 min. The supernatant was stored at −20°C for later use.

### Reagents and equipment

Rabbit anti-human somatostatin (SS) and vasoactive intestinal peptide (VIP) secondary antibodies, and a 3,3′-diaminobenzidine (DAB) kit were obtained from Beijing Zhongshan Biotechnology Co., Ltd. (Beijing, China). The radioimmunoassay kits for SS and VIP were purchased from Beijing Huaying Institute of RIA Technology (Beijing, China), while a Motic Med 6.0 Digital Medical Image Analysis system was obtained from Motic China Group Co., Ltd. (Xiamen, China). A CV-70 Colonoscope was purchased from the Olympus Corporation (Tokyo, Japan), and an SN-695B Smart Radioimmunoassay Analyzer was obtained from the first Rihuan Equipment Factory (Shanghai Institute of Atomic Nuclear Research, Shanghai, China).

### Radioimmunoassay

The concentrations of SS and VIP in the blood and sigmoid colon mucosa were measured in the SN-695B radioimmunoassay analyzer (The First Rihuan Equipment Factory, Shanghai, China), using a sequence saturation method. The standard curve was automatically drawn.

### Immunohistochemical analysis

#### Preparation of paraffin sections

The sigmoid colon tissue was rapidly placed into 10% formalin for 10 h of fixation at 4°C, and then a gradient dehydration was performed (80% alcohol, 12 h; 95% alcohol, 2 h; anhydrous alcohol I, 1 h and anhydrous alcohol II, 1 h). Following this, the tissue was immersed in xylene for 1 h, and then in paraffin I and paraffin II at 62°C for 1 h, respectively. Subsequent to dehydration, the sample was successively immersed in liquid 1 (embedding agent:acetone, 1:3) for 1 h, liquid 2 (embedding agent:acetone, 1:1) for 1.5 h, liquid 3 (embedding agent:acetone, 3:1) for 2 h and then liquid 4 (embedding agent) for 5 h (or overnight). The sample was added to the bottom of embedding capsule using a toothpick, and the sample label was placed in the capsule. Following this, the embedding agent was added and polymerization was performed at 60°C for 48 h. The polymerized embedding block was coarsely trimmed using a trimming machine, and then manually trimmed in an Ultracut ultramicrotome (Leica Microsystems, Wetzlar, Germany). Subsequent to positioning, the embedding block was sliced into sections with a thickness of 5 μm, followed by seeding onto poly-L-lysine-coated glass slides. The sections were then dried overnight at 60°C.

#### Immunohistochemical staining

The sections were immersed in xylene three times (5 min each time), and then in absolute alcohol, 95% alcohol, 80% alcohol and 70% alcohol for 3 min, respectively, to remove the residual xylene. Following this, the sections were hydrated using double distilled water (or tap water), and then incubated in 0.3% methanol-hydrogen peroxide at room temperature for 20 min. Subsequently, the sections were washed three times with distilled water and antigen retrieval was performed using 0.1 M citric acid buffer (pH 6) in a microwave oven (98°C) for 20 min. Once the sections had cooled to room temperature, 50 μl goat serum was added to each of the sections, which were then incubated at 37°C for 30 min. The excess serum was drained using filter paper, prior to the primary antibody (anti-SS, 1:50 or anti-VIP, 1:700) being added, and the overnight incubation of the sections at 4°C. Phosphate-buffered saline (PBS) was added to the negative control group, instead of primary antibody. Following the removal of excess primary antibody, the sections were washed with 0.01 M PBS three times, and the excess PBS was removed. Biotin-conjugated secondary antibody (50 μl) was added to the sections, which were then incubated for 30 min at 37°C, prior to being washed three times with 0.01 M PBS. Following the removal of excess PBS, horseradish peroxidase (HRP)-labeled avidin was added to the sections, which were subsequently incubated for 30 min at 37°C. The sections were then washed three times using 0.01 M PBS. Subsequent to the removal of excess PBS, 100 ml DAB and one drop of 3% H_2_O_2_ were added for DAB staining for 2–5 min, prior to the staining being terminated by washing with tap water. Hematoxylin staining was then performed. Following a gradient dehydration (70% alcohol, 5 min; 80% alcohol, 5 min; 90% alcohol, 5 min; anhydrous alcohol I, 15 min and anhydrous alcohol II, 15 min), the sections were successively immersed in xylene I, xylene II and xylene III for 15 min, and were then fixed with neutral gum.

#### Statistical analysis

Statistical analysis was performed with SPSS software, version 13.0 (SPSS, Inc., Chicago, IL, USA). All data are presented as the mean ± standard deviation, and were compared using a two sample t-test. P<0.05 was considered to indicated a statistically significant difference.

## Results

### Mental condition of the patients

A significant difference was observed between the anxiety-depression scores of the IBS and the control groups (P<0.05; Table I).

### Protein expression of SS and VIP in the blood and colonic mucosa

The protein expression levels of SS in the blood and colonic mucosa of the patients with IBS were increased significantly compared with those of the control patients (P<0.05). Moreover, the expression levels of SS in the blood and colonic mucosa of the IBS-C group demonstrated significantly larger increases than those in the IBS-D group (P<0.05). The expression levels of VIP protein in the blood and colonic mucosa of the patients with IBS were significantly increased compared with those of the control patients (P<0.05); however, there were not observed to be any significant differences between the VIP levels of the IBS-C and IBS-D groups (P>0.05; Table II).

### Differences between the protein expression of SS and VIP in the blood and colonic mucosa of the IBS-C group

Patients with IBS-C in anxiety and depressive states expressed significantly higher levels of SS and VIP in the serum and colonic mucosa than IBS-C patients with normal anxiety and depression ratings (P<0.05; Table III).

### Differences between the protein expression of SS and VIP in the blood and colonic mucosa of the IBS-D group

There was a significantly greater increase in the levels of protein expression of SS and VIP in the blood and colonic mucosa of the patients with IBS-D with anxiety and depression, in comparison with those with IBS-D and normal anxiety and depression ratings (P<0.05; Table IV).

### Protein expression of SS and VIP in the colonic mucosa, detected by immunohistochemistry (IHC)

The morphological expression of the SS and VIP proteins in the colonic mucosa of patients was further examined using immunohistochemical staining techniques. The results demonstrated that there were significant increases in the numbers of SS^+^ and VIP^+^ cells in the sigmoid colon mucosa of the patients with IBS, compared with the controls. Furthermore, there was a marked increase in the number of SS^+^ cells in the IBS-C group, compared with the IBS-D group (P<0.05). However, no significant difference was observed between the IBS-C and IBS-D groups, with regard to the number of VIP^+^ cells (Table V).

### SS^+^ and VIP^+^ cells in the colonic mucosa of the patients with IBS-C, with different SAS and SDS scores

Significant differences were observed between the numbers of SS^+^ and VIP^+^ cells in the colonic mucosa of the patients in the IBS-C group with anxiety and depression, in comparison with the numbers in the patients in the IBS-C group with normal anxiety and depression ratings (P<0.05; Table VI).

### SS^+^ and VIP^+^ cells in the colonic mucosa of the patients with IBS-D, with different SAS and SDS scores

An analysis of the numbers of SS^+^ and VIP^+^ cells in the colonic mucosa of the patients with IBS-D with anxiety and depression and of those with normal anxiety and depression ratings revealed that there were significantly greater numbers of SS^+^ and VIP^+^ cells in the patients with anxiety and depression than in the patients with normal emotional state ratings (P<0.05; Table VII).

## Discussion

IBS is characterized by a group of symptoms, including abdominal pain or bloating, and abnormal bowel habits and stool consistency. It is a persistent or intermittent syndrome that lacks any obvious morphological changes and biochemical abnormalities (1,2). In normal circumstances, the body is regulated by the nerve-endocrine network and immune system. However, when pathogenic factors are apparent, visceral sensation and gastrointestinal smooth muscle dyskinesia occur, and eventually lead to the onset of IBS (8,9). Gastrointestinal hormones and psychological factors are important in the pathophysiological process of IBS.

The regulation of psychological factors and gastrointestinal functions, such as movement and feeling, may be achieved through the nerve-endocrine-immune network (10). Negative emotions, such as anxiety and depression, lead to the cerebral cortex and limbic system becoming disturbed. This affects the function of the enteric nervous system through the brain-gut axis neurohumoral system, and leads to abnormalities in gastrointestinal hormone secretion. In addition, it leads to colonic epithelial barrier and mucosal immune dysfunction, as well as the reinforcement of colonic movement and visceral sensitivity. Consequently, it results in changes in gastrointestinal functions, leading to diarrhea, constipation or abdominal pain, and leads to the occurrence of IBS (11).

SS suppresses the secretion of gastrointestinal hormones and gastric acid, and inhibits the movement of gastrointestinal and biliary smooth muscle. It also reduces the intestinal absorption of water, electrolytes and nutrients. In this study, it was observed that the expression of SS in the blood, colonic mucosa and sigmoid colonic mucosa in patients with IBS was higher than in the normal control group, and that there was a significantly larger increase in the IBS-C group than in the IBS-D group. The results indicated that there was an abnormal secretion of SS in the patients with IBS, which concurred with the findings of previous studies. The high level of secretion of SS in the IBS-C group indicated that constipation may result from the suppression of gastrointestinal functions by SS. However, for the IBS-D group, the increment in SS may have been insufficient to suppress the augmented intestinal movement and, therefore, symptoms such as abdominal pain and diarrhea were observed (8,12,13).

The biological function of VIP is the suppression of the tension of the lower esophageal sphincter and the reduction of gastrointestinal muscular tension, in addition to inhibition of the contraction of the gallbladder and the intestinal absorption of water and electrolytes (14,15). The present study demonstrated that the expression of VIP increased significantly in the blood, colonic mucosa and sigmoid colonic mucosa of the patients with IBS, compared with the control group. However, there was no significant difference between the IBS-C and IBS-D groups. The results suggested that VIP may be involved in different stages of the pathogenesis of IBS. In the patients with IBS-C, the increase in VIP reduced the peristaltic contractions and led to constipation, whereas in the patients with IBS-D, the high secretion of VIP promoted the secretion of intestinal water and electrolytes, and led to diarrhea. It was observed by Ringel *et al* that there was an elevated expression of VIP in the blood and sigmoid colonic mucosa of patients with IBS-C; however, a contrasting result was revealed in the patients with IBS-D (16). A possible mechanism of action may be that the increase in VIP levels in the patients with IBS-C resulted in the inhibition of intestinal motility and a reduction in the speed of peristaltic contractions, ultimately leading to constipation, or, otherwise, leading to IBS-D. This suggested that abnormal neurohumoral regulation does exist in the pathogenesis of IBS. However, these conclusions are different from those of other studies, which have indicated that the pathogenesis of IBS is too complicated to be elucidated. The interactions involved and their impacts remain to be determined.

In the current study, it was observed that the SAS and SDS scores were significantly different between the IBS and control groups. Furthermore, the expression levels of SS and VIP in the blood, colonic mucosa and sigmoid colonic mucosa were higher in the patients with IBS in anxiety and depressive states than in those with normal emotional state ratings. The results indicated that the onset or aggravation of IBS is associated with psychological factors, which increase the susceptibility to IBS, and promote the development and maintenance of the condition. It has been revealed that the interrelation between the severity of the IBS and psychosocial factors is more significant than with physiological factors (17–20). It has also been observed that the abnormal psychology in IBS is closely correlated with a further critical etiological factor, gastrointestinal hormones (21–23). Abnormal mental states of anxiety and depression may change the regulatory functions of the autonomic nervous system center and the neuroendocrine center of accommodation, and transfer the message to the enteric nervous system, leading to an alteration in gastrointestinal hormones such as SS and VIP. This may influence visceral sensation, intestinal movement and endocrine function, and lead to the onset or aggravation of symptoms in the digestive tract of the patient with IBS. Conversely, the chronic presence of somatic symptoms increases the psychological burden of the patients and exacerbates the anxiety and depression experienced. There is therefore an interrelation between the psychological burden of patients with IBS and the IBS itself, with each factor influencing and promoting the other (24,25).

The etiological factors in IBS are exceedingly complicated, and there is no accepted theory to explain the pathogenesis of the disease. Therefore, further investigations into the etiology and pathogenesis of IBS are required, in order to provide a direct interventional target for the treatment of IBS. In this manner, the diagnosis and treatment of IBS may be improved.

## Figures and Tables

**Table I:** Comparison between the anxiety and depression scores in the IBS and control groups.

Group	SDS	SAS
Control	34.12±10.30	37.65±11.97
IBS	40.98±11.76^a^	48.32±14.32^a^

Data are presented as the mean ± standard deviation.

a P<0.05 versus the control group.

IBS, irritable bowel syndrome; SDS, Self-Rating Depression Scale; SAS, Self-Rating Anxiety Scale.

**Table II:** Protein expression levels of SS and VIP in the blood and colonic mucosa.

	SS (ng/l)		VIP (ng/l)	
		
Group	Blood	Sigmoid colon	Blood	Sigmoid colon
Control	27.43±18.45	18.21±6.24	35.81±19.30	725.43±179.23
IBS-C	170.23±38.47^a^	256.20±178.41^a^	51.73±29.53^a^	1,234.07±472.83^a^
IBS-D	59.82±20.63^a^^b^	35.73±17.47^a^^b^	53.34±21.64^a^^c^	1,210.75±384.73^a^^c^

Data are presented as the mean ± standard deviation.

a P<0.05 versus the control group;

b P<0.05 versus the constipation predominant irritable bowel syndrome (IBS-C) group;

c P>0.05 versus the IBS-C group.

SS, somatostatin; VIP, vasoactive intestinal peptide. IBS-D, diarrhea predominant IBS.

**Table III:** Protein expression levels of SS and VIP in the blood and colonic mucosa of IBS-C patients.

	SS (ng/l)		VIP (ng/l)	
		
Group	Blood	Sigmoid colon	Blood	Sigmoid colon
IBS-C with normal emotional state ratings	149.67±32.45	231.45±172.93	48.52±31.69	1,058.24±398.73
IBS-C in anxiety-depressive states	175.79±45.21^a^	254.19±174.28^a^	62.13±30.14^a^	1,295.39±372.76^a^

Data are presented as the mean ± standard deviation.

a P<0.05 versus the constipation predominant irritable bowel syndrome (IBS-C) with normal emotional state ratings group.

SS, somatostatin; VIP, vasoactive intestinal peptide.

**Table IV:** Protein expression levels of SS and VIP in the blood and colonic mucosa of IBS-D patients.

	SS (ng/l)		VIP (ng/l)	
		
Group	Blood	Sigmoid colon	Blood	Sigmoid colon
IBS-D with normal emotional state ratings	40.67±21.77	30.56±20.83	46.48±21.01	1,021.44±201.56
IBS-D in anxiety-depressive states	60.43±19.23^a^	35.90±21.96^a^	60.69±19.23^a^	1,277.92±177.38^a^

Data are presented as the mean ± standard deviation.

a P<0.05 versus the diarrhea predominant irritable bowel syndrome (IBS-D) with normal emotional state ratings group.

SS, somatostatin; VIP, vasoactive intestinal peptide.

**Table V:** Morphological expression of SS and VIP in the colonic mucosa of patients.

Group	SS^+^ (ng/l)	VIP^+^ (ng/l)
Control	0.075±0.014	0.089±0.021
IBS-C	0.256±0.090^a^	0.324±0.125^a^
IBS-D	0.164±0.131^a^^b^	0.358±0.220^a^^c^

Data are presented as the mean ± standard deviation.

a P<0.05 versus the control group;

b P<0.05 versus the constipation predominant irritable bowel syndrome (IBS-C) group;

c P>0.05 versus the IBS-C group.

SS, somatostatin; VIP, vasoactive intestinal peptide; IBS-D, diarrhea predominant IBS.

**Table VI:** SS^+^ and VIP^+^ cells in the colonic mucosa of IBS-C patients, detected by immunohistochemistry.

Group	SS^+^ (ng/l)	VIP^+^ (ng/l)
IBS-C with normal emotional state ratings	0.213±0.061	0.259±0.159
IBS-C in anxiety-depressive states	0.285±0.113^a^	0.374±0.125^a^

Data are presented as the mean ± standard deviation.

a P<0.05 versus the constipation predominant irritable bowel syndrome (IBS-C) with normal emotional state ratings group.

SS, somatostatin; VIP, vasoactive intestinal peptide.

**Table VII:** SS^+^ and VIP^+^ cells in the colonic mucosa of IBS-D patients, detected by immunohistochemistry.

Group	SS^+^ (ng/l)	VIP^+^ (ng/l)
IBS-D with normal emotional state ratings	0.143±0.101	0.275±0.178
IBS-D in anxiety-depressive states	0.207±0.063^a^	0.400±0.220^a^

Data are presented as the mean ± standard deviation.

a P<0.05 versus the diarrhea predominant irritable bowel syndrome (IBS-D) with normal mental state ratings group.

SS, somatostatin; VIP, vasoactive intestinal peptide.
